# First tracking of the oceanic spawning migrations of Australasian short-finned eels (*Anguilla australis*)

**DOI:** 10.1038/s41598-021-02325-9

**Published:** 2021-11-26

**Authors:** Wayne M. Koster, Kim Aarestrup, Kim Birnie-Gauvin, Ben Church, David Dawson, Jarod Lyon, Justin O’Connor, David Righton, Denis Rose, Håkan Westerberg, Ivor Stuart

**Affiliations:** 1 Department of Environment, Land, Water and Planning, Arthur Rylah Institute for Environmental Research, 123 Brown Street, Heidelberg, VIC 3084 Australia; 2 Technical University of Denmark, Vejlsøvej 39, 8600 Silkeborg, Denmark; 3 Gunditj Mirring Traditional Owner Aboriginal Corporation, Edgar Street, Heywood, VIC 3304 Australia; 4 Centre for Environment, Fisheries and Aquaculture Science, Pakefield Road, Lowestoft, NR330HT Suffolk UK; 5 School of Environmental Sciences, University of East Anglia, Norwich, NR4 7TJ UK; 6 Department of Aquatic Resources, Institute of Freshwater Research, Swedish University of Agricultural Sciences, Stångholmsvägen 2, 178 93 Drottningholm, Sweden

**Keywords:** Animal migration, Animal behaviour, Conservation biology

## Abstract

Anguillid eel populations have declined dramatically over the last 50 years in many regions of the world, and numerous species are now under threat. A critical life-history phase is migration from freshwater to distant oceans, culminating in a single life-time spawning event. For many anguillids, especially those in the southern hemisphere, mystery still shrouds their oceanic spawning migrations. We investigated the oceanic spawning migrations of the Australasian short-finned eel (*Anguilla australis*) using pop-up satellite archival tags. Eels were collected from river estuaries (38° S, 142° E) in south-eastern temperate Australia. In 2019, 16 eels were tracked for up to about 5 months, ~ 2620 km from release, and as far north as the tropical Coral Sea (22° S, 155° E) off the north-east coast of Australia. Eels from southern Australia appeared to access deep water off the Australian coast via two main routes: (i) directly east via Bass Strait, or (ii) south-east around Tasmania, which is the shortest route to deep water. Tagged eels exhibited strong diel vertical migrations, alternating between the warm euphotic zone (~ 100–300 m, 15–20 °C) at night and the mesopelagic zone (~ 700–900 m, 6–8 °C) during the day. Marine predators, probably lamnid sharks, tuna, or marine mammals, ended many eel migrations (at least ~ 30%), largely before the eels had left the Australian continental shelf. The long and risky marine migrations of Australasian eels highlight the need for better information on the processes contributing to eel mortality throughout the life cycle, including the impacts of future changes to oceanic currents, predator abundance and direct anthropogenic disturbances.

## Introduction

Worldwide, many migratory fish species are threatened with extinction due to human activities^1–3^. Diadromous species migrate between marine and estuarine or freshwater habitats as part of their life cycle, often over hundreds or thousands of kilometres at multiple life stages, which presents major risks to survival^4^. The spatial scale and diversity of the marine, estuarine, and freshwater habitats over which diadromous species migrate exacerbates the anthropogenic stressors, which increases population vulnerability and raises significant challenges for conservation^5^. Among diadromous fishes, anguillid eels are particularly at risk, with 6 of the 19 species or subspecies (4 temperate and 2 tropical species) listed within IUCN Threatened categories^6^; www.iucnredlist.org).

Anguillid eels are globally distributed in temperate, tropical, and subtropical areas. A critical stage in their life cycle is the migration of mature adults from freshwater habitats and estuaries to tropical marine spawning grounds (i.e. catadromy)^7^. The oceanic spawning migrations of a few (primarily northern hemisphere) species, such as the European eel (*Anguilla anguilla*)^8,9^, Japanese eel (*Anguilla japonica*)^10^, and American eel (*Anguilla rostrata*)^11^, have recently been investigated using pop-up satellite tags (PSATs) resulting in significant new information on migration patterns and behaviour. However, there is little information on the adult marine migration of southern hemisphere anguillids, especially in temperate regions (but see articles by Jellyman and Tsukamoto^12^ and Watanabe et al.^13^).

The Australasian short-finned eel (*Anguilla australis*) is a catadromous, semelparous species found in eastern Australia (including Norfolk Island and Lord Howe Island), New Zealand, and New Caledonia. Short-finned eels are harvested in both commercial and recreational fisheries in Australia and New Zealand and have a particular cultural significance to First Nations people. For example, the Gunditjmara people of south-western Victoria built and used sophisticated aquaculture systems throughout the Budj Bim cultural landscape to exploit eel migrations at least 7000 years ago^14^. These systems and their eel catches have since provided a lasting and sustainable economic and social base for the Gunditjmara society^15^. The Budj Bim landscape was recently inscribed on the World Heritage list as one of the world’s most extensive and oldest aquaculture systems (https://whc.unesco.org/en/list/1577/). Australasian short-finned eels are listed as ‘near threatened’ on the IUCN Red List of Threatened Species^16^, with barriers to riverine movement and freshwater habitat loss being key threats. In addition, changes in ocean currents, primary production, and thermal regimes may also affect eel migration, spawning success, and recruitment^17,18^.

The oceanic migratory routes, timing, and precise spawning locations of adult short-finned eels are largely unknown. Schmidt^19^ originally hypothesized that short-finned eels from Australia spawned on the western side of the New Caledonian submarine ridge, and fish from New Zealand spawned on the eastern side of the ridge. Since then, small numbers of leptocephali have been collected in widely separated areas of the Solomon Islands, Vanuatu, New Caledonia, and Fiji, on both sides of the Vanuatu Archipelago^20–23^. Our aim was to use PSATs to directly examine for the first time the oceanic migrations of adult short-finned eels from Australia, including their vertical movement behaviour, migration routes, response to the lunar cycle and predation.

## Methods

### Fish collection and tagging

**Figure 1:**
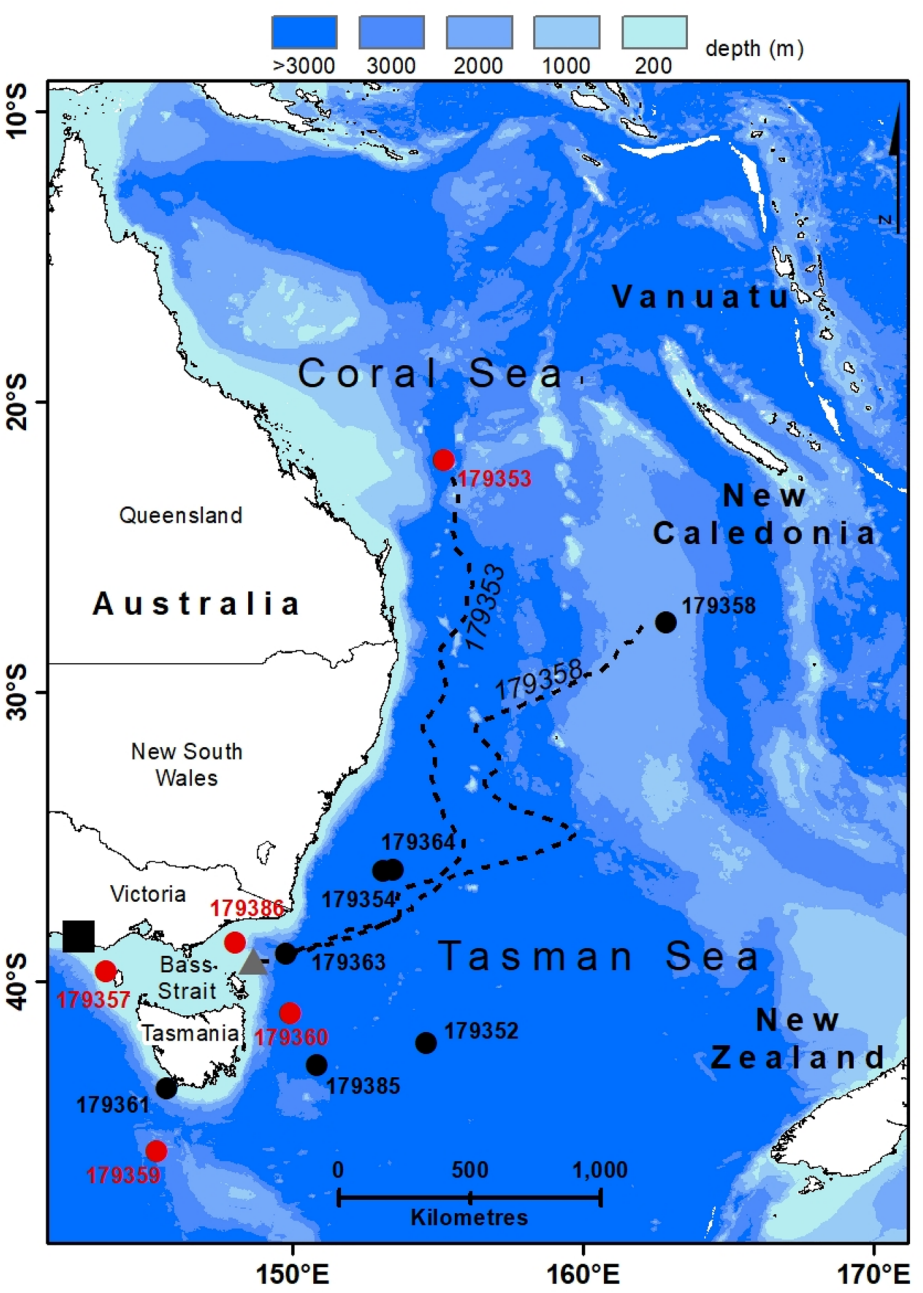
Map showing location of the study area. Black square denotes tagging location. Grey triangle denotes approximate exit point from Bass Strait for eels leaving towards the east. Black circles denote end positions of tags with premature ending and sudden rise to the surface. Red circles denote end positions where eels were inferred to be predated. The dashed black lines shows the approximate trajectories of eel 179353 and eel 179358. Figure produced using ESRI ArcGIS 10.7.1 (https://www.esri.com/en-us/arcgis/products/arcgis-desktop/overview).

Sixteen adult short-finned eels (mean total length [TL] 979 mm, range 900–1070 mm; mean weight 1906 g, range 1606–2450 g) were collected from the mouths of the Hopkins and Fitzroy river estuaries (38° S, 142° E) (Fig. 1) in April 2019. The eels were collected, using either fyke nets or dip nets, as they migrated from the river mouths outwards to the Southern Ocean over a sandbar periodically inundated by waves (Table 1). Only eels larger than 900 mm, all of which were likely female^24,25^, were retained for tagging.

**Table 1:** Biological data for tagged adult female short-finned eels from south-western Victoria, Australia. All eels were collected as they migrated out of estuarine water.

Date	Tag ID	Length (mm)	Weight (g)	Capture location	Release location	PSAT scheduled pop-up time (months)
17/04/2019	179352	1037	1630	Hopkins River mouth	Hopkins Mouth Beach	6
17/04/2019	179353	990	1810	Hopkins River mouth	Hopkins Mouth Beach	6
24/04/2019	179354	970	1792	Hopkins River mouth	Warrnambool harbour	6
24/04/2019	179355	940	1824	Hopkins River mouth	Warrnambool harbour	6
25/04/2019	179356	990	2150	Hopkins River mouth	Killarney Beach	6
26/04/2019	179357	980	2050	Fitzroy River mouth	Warrnambool harbour	6
26/04/2019	179358	1020	2050	Hopkins River mouth	Warrnambool harbour	6
17/04/2019	179359	990	1690	Hopkins River mouth	Warrnambool harbour	7
24/04/2019	179360	900	1730	Hopkins River mouth	Warrnambool harbour	7
24/04/2019	179361	946	1744	Hopkins River mouth	Warrnambool harbour	7
26/04/2019	179362	1070	2394	Hopkins River mouth	Warrnambool harbour	7
26/04/2019	179363	1030	2450	Hopkins River mouth	Warrnambool harbour	7
26/04/2019	179364	920	1854	Hopkins River mouth	Warrnambool harbour	7
17/04/2019	179385	960	1740	Hopkins River mouth	Warrnambool harbour	8
24/04/2019	179386	1000	1980	Hopkins River mouth	Warrnambool harbour	8
24/04/2019	179387	920	1606	Hopkins River mouth	Warrnambool harbour	8

For tagging, eels were transferred into an aerated 150 L holding container of estuarine water and then individually anaesthetised (0.09 mL AQUI-S per litre water) (AQUI-S, Lower Hutt, New Zealand). Time to anaesthesia was ~ 8–10 min. The PSAT tags (Microwave Telemetry X-tag http://www.microwavetelemetry.com/) were attached externally using stainless steel wires in a 3-point attachment inserted dorsally under the skin, as per the Westerberg method^26^. The tags were 120 mm long, with a 185 mm long antenna, a maximum float diameter of 33 mm, and a mass of 45 g in air. The tags are weakly buoyant and float in water^8^, and they are coated with an anti-fouling paint. Throughout the procedure, the head and gills of the eels were immersed in aerated anaesthetic solution. Each surgery, including measurements, took ~ 6 min. The fish were then placed into a 150 L recovery container filled with estuarine water. Once the eels were observed to maintain equilibrium and swim freely, they were released from the holding container into a nearby sheltered ocean harbour or beach.

The PSATs were set to release on a specific day, 6, 7, or 8 months after deployment, the hypothesized time required for migration to the spawning area^27^. The PSATs also had a built-in mortality option so that the tag would release from the eel, float to the surface and start transmitting data if a constant pressure was recorded for a user-defined period of time. The purpose of this option was to retrieve the data as quickly as possible if a tagged fish died, but because eels often rest buried on the seabed immediately after release, the required minimum period was set to 4 days to avoid accidental early release. However, a consequence of this was that if the tag was separated from the eel before the programmed date and started drifting at the sea surface, there was a delay of 4 days before the start of transmission.

### Mapping migrations

Light-based geolocation methods are not possible for anguillid eels because, unlike surface-oriented fish species, they typically occupy oceanic depths beyond the capability of tag-mounted light sensors. However, because anguillid eels exhibit a strong diel vertical migration (DVM) driven by changes in light intensity^28^, methods have now been established that enable the calculation of estimates of longitude by determining the time of local noon from the large observed changes in depth or temperature that occur when eels ascend at dusk or descend at dawn in the water column^29^. For our *A. australis* data, estimates of local noon were calculated in a similar manner to that described by Chang et al.^30^ for *Anguilla marmorata,* because of the similarity in the depth ranges and slight asymmetry in the timings of the dusk ascent and dawn descent. However, because a significant proportion of the depth and temperature data collected during crepuscular periods for *A. australis* could not be used because it was ‘delta-limited’ (a data compression technique used in X-tags, for which true depth differences between measurements are replaced by a value limited by the digitization of the actual measurement values), a simplified version of the Chang et al. method^30^ was used, which is detailed in the Supplementary Information. Similar methods using the timing of DVM to estimate dawn and dusk, and therefore calculate estimates of longitude, have been used in studies of other anguillid eel species (e.g., European eel^31^) and are now well established. To reconstruct a complete oceanic movement trajectory of an eel, we assumed that eels maintained a constant swimming speed, and latitude was therefore able to be calculated for each point, based on the overall direction of travel (see “Results” for details).

To examine the influence of moon phase on movement behaviour, the mean night-time swimming depth was calculated daily for the time interval 22:00–01:00 h for the two eels with the longest tracks (179353 and 179358). The mean night-time swimming depth was plotted as a function of the integer value of the age of the moon. The moon phase was calculated with AstroExcel (https://astroexcel.wordpress.com/). The time of full moon occurs at a moon age between day 14 and 15.

All field work was performed in accordance with the relevant guidelines and regulations under Victorian Flora and Fauna Guarantee Permit 10005451 and Fisheries Victoria Research Permit RP-827. This study was approved and conducted under ethics permit 18-006 (Arthur Rylah Institute for Environmental Research Animal Ethics Committee). Reporting in the manuscript follows the recommendations in the ARRIVE guidelines.

## Results

### Data recovery

Of the 16 eels tagged, data was obtained from 12 (Fig. 1, Table 2); time between release and surfacing of the tag ranged from 7 to 140 days (average 41.8 ± 39.6 SD). All tags were released prematurely from the eels, presumably because of ingestion by marine predators (see below) or failure of the attachment. Eleven tags transmitted data via the Argos system (https://www.argos-system.org/), and the data return was generally high (76–98%).

**Table 2:** Track details for tagged adult female short-finned eels from south-western Victoria in 2019.

		179352	179353	179354	179357	179358	179359	179360	179361	179363	179364	179385	179386
Scheduled pop-up time	days	180	180	180	180	180	210	210	210	210	210	240	240
Actual pop-up time	days	38	NA	30	17	146	22	22	12	51	62	45	15
Total track	km	1060	2620	1010	120	2320	810	700	640	1450	1830	1100	485
Max depth	m	1016	1120	1020	107	1020	905	75	97	1055	1065	1280	72
Min temp	°C	6.0	4.7	6.9	15.0	4.4	6.6	16.4	16.0	6.1	4.4	4.2	16.5
Max temp	°C	22.5	37.1	20.6	26.2	26.4	37.1	26.7	17.1	17.1	19.9	15.9	26.7
Release	Date	17 April	17 April	24 April	26 April	26 April	17 April	24 April	24 April	26 April	26 April	17 April	24 April
	Long E	142.51	142.51	142.48	142.48	142.48	142.48	142.48	142.48	142.48	142.48	142.48	142.48
	Lat N	− 38.40	− 38.40	− 38.40	− 38.40	− 38.40	− 38.40	− 38.40	− 38.40	− 38.40	− 38.40	− 38.40	− 38.40
First Argos location	Date	25 May		24 May	13 May	19 Sept	9 May	16 May	6 May	16 June	27 June	1 June	9 May
	Long E	154.72		152.99	144.26	162.08	145.94	146.23	145.60	149.80	153.38	149.63	148.25
	Lat N	− 33.59		− 36.64	− 39.65	− 17.06	− 43.61	− 38.91	− 39.42	− 38.64	− 37.08	− 42.63	− 38.37
Sun geolocation	Date	12 May	18 July	23 May	9 May	13 Sept	5 May	9 May	1 May	13 June	24 June	1 June	5 May
	Long E	154.60	155.20	153.12	143.57	162.87	145.32	149.90	145.64	149.77	153.45	150.84	148.02
	Lat N	− 42.10	− 22.00	− 36.16	− 39.63	− 27.59	− 45.82	− 41.08	− 43.65	− 39.01	− 36.13	− 42.87	− 38.66
Migration on shelf	Route direction	East	East	East		East	South			South	South	South	
	End date	27 April	28 April	6 May		10 May	19 April			30 April	29 April	19 April	
	Duration days	10.2	11.3	12.2		14.3	1.5			4.4	3.3	2.3	
	Distance km	480	480	480		480	120			120	120	120	
	km/day	47.2	42.6	39.2		33.5	80.0			27.6	35.9	51.2	
Diel vertical migration	Long E (start of DVM)	148.5	149.0	153.0		149.5	145.5			144.5	147.0	143.0	
	Start date	27 April	28 April	6 May		10 May	19 April			30 April	29 April	19 April	
	End date	9 May	17 July	22 May		14 Sept	6 May			14 June	25 June	1 June	
	Duration days	11.6	79.6	15.9		126.6	17.0			44.8	56.4	103.6	
	Distance km	580	2120	530		1840	690			1330	1710	980	
	km/day	49.8	26.6	33.2		14.5	40.6			29.7	30.3	9.5	
Sun to first Argos location	Duration days	12.7		1.5	3.8	6.4	4.2	6.8	4.8	3.3	2.8	0.1	4.2
	Distance km	947		55	59	1153	251	399	471	41	106	102	38
	Speed km/day	74.3		37.8	15.4	184.9	59.7	58.6	97.8	12.4	38.0	na	9.1
Ending		Dives from surface	Whale predation	Abrupt end	Predation	Dives from surface	Whale predation	Predation	Dives from surface	Abrupt end	Abrupt end	Too deep	Predation

*na* Not applicable.

As all tags detached prematurely, transmission of data began 4 days after release as a consequence of the constant-depth mortality fail-safe, and therefore they may have drifted a considerable distance from the final position of the eel. For this reason, and because the tags collected light data at the sea surface while drifting, the light-based geolocation estimate provided by the PSAT from the first day after release was used as the final position of the eel, rather than the first transmitted Argos location (median drift displacement = 106 km, range 38–1153 km; Table 2). Tags popped up to the south, east, and the north-east of the release location. Distance between the release location and the final position ranged from 120 to 2620 km (Fig. 1, Table 2), expressed as the approximate distance over water. For eels that left the shelf directly south of the release point, the distance was measured around Tasmania. One of the four tags (179353) that did not transmit data was found at Palfrey Island (14° S, 145° E), part of the Lizard Island Group, 270 km north of Cairns, with a broken antenna; data was extracted from the tag memory by the manufacturer.

### Vertical movement behaviour

**Figure 2:**
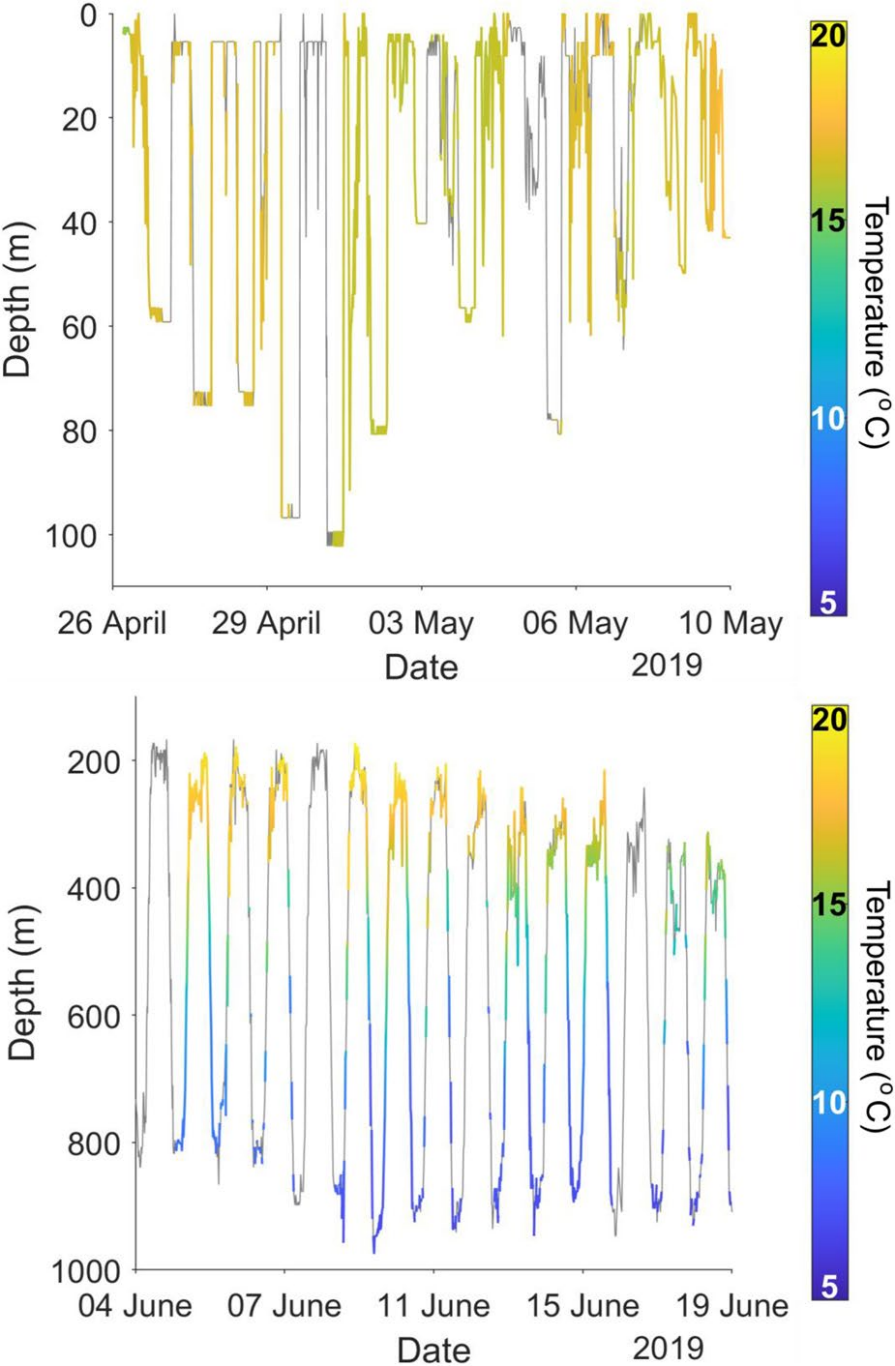
Example of a short-finned eel (179358) remaining in shallow water on the Australian continental shelf during the first days following tagging (top panel), followed by regular diel vertical migration, moving from shallower to deeper water between night and day once in deep water (bottom panel), showing depth coloured by temperature (°C). Note: grey denotes where a corresponding temperature was not available for a depth measurement.

**Figure 3:**
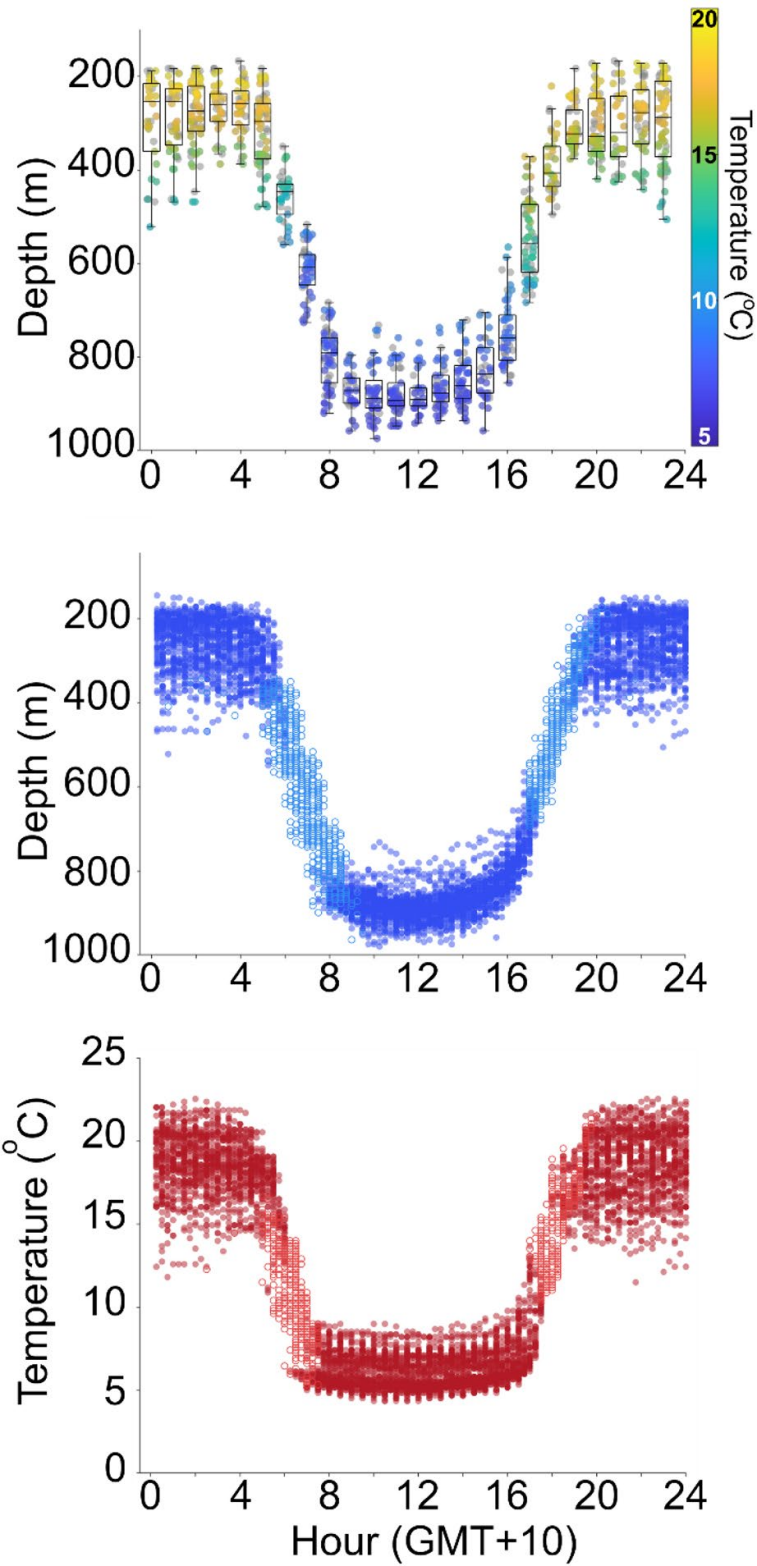
Diel temperature and depth data from 179358. For top panel, boxes show the median (midline), lower and upper quartiles (lower and upper limits of the box), and the extent of any outliers (whiskers); scatter points show depth coloured by temperature; and grey points show where a corresponding temperature was not available for a depth measurement. For middle and lower panel, delta-limited values are shown by hollow symbols.

Eels remained in shallow water (< 200 m deep) for up to 14 days after release, while they moved east or south along the Australian continental shelf. During this time, the diving behaviour was irregular. Eels typically stayed at a steady depth at the presumed sea floor during daylight and ascended toward the sea surface at night (Fig. 2). Eels were often active during the night, diving away from and returning to the sea surface, until descending at dawn to the seabed. Once they reached the edge of the continental shelf and gained access to deep water (> 200 m), all eels exhibited a DVM (Fig. 2; Fig. 3), which typified their oceanic migration phase. This DVM consisted of alternating between occupation of the warm euphotic zone (~ 100–300 m, 15–20 °C) at night, and occupation of the mesopelagic zone (~ 700–900 m, 6–8 °C) during the day (Fig. 3). The transition between these two ocean layers occurred at dusk and dawn each day, when eels dived or ascended rapidly in a near-continuous movement to the day-time or night-time depths. All eels made occasional excursions below 1000 m depth, except eel 179359, which had a maximum dive to 905 m. The deepest depth recorded was by eel 179385 which reached 1280 m, a depth that triggered the fail-safe depth release of the PSAT and ended the deployment prematurely. The average temperature experienced at night-time increased as eels moved north toward the Coral Sea, but temperatures during the day remained similar throughout the migration, irrespective of location.

### Migration routes

Tags from four eels did not record data for longer than 14 days and detached before the eels reached the Tasman Sea. The remaining eight eels were tracked into the Tasman Sea beyond the Australian continental shelf, reaching deep water (i.e., > 500 m depth) either (i) between 2 and 4 days, or (ii) between 10 and 14 days, after release (Fig. 1; Table 2). On the basis of time taken to reach deep water, and estimates of longitude at the beginning of DVM, these results indicate that the eels accessed deep water off the Australian coast: (i) by swimming south-east and circumnavigating Tasmania, or (ii) by swimming directly east through Bass Strait, respectively. The average speed over ground was 30.8 ± 7.3 km/day while eels were on the continental shelf (Table 2).

Once in deep water over the continental slope, the eight eels exhibited regular DVM. This DVM was maintained for between 12 and 127 days and, for seven of the eels, was maintained until tag surfacing, indicating that these eels were travelling under their own volition before the tag detached or the eel was predated. Two tags (179359, 179385) surfaced near Tasmania (one south-west and one south-east), one off the eastern Victorian coast (179363), and two (179354, 179364) off the southern New South Wales coast. Tags from two of these eels (179353, 179358) surfaced well off the Queensland coast in the tropical Coral Sea (179353 at 22° S, 155° E north of the Tropic of Capricorn; Fig. 1). The DVM of one eel (179352) ceased about 4 days before the tag surfaced well off the eastern coast of Tasmania, so it is unclear whether the eel swam or the detached tag drifted to the final position. Overall, eels maintained an average speed over ground of 29.7 ± 11.1 km/day while in deep water.

A simplified reconstruction of the longest two migrations (179353, 178358) was made by assuming a constant swimming speed and constant daily latitude increment between the beginning and end of the active migration (Fig. 1). Eel 179358 travelled eastwards and north after exiting the Bass Strait, with a number of longitudinal meanders. The trajectory for eel 179353 was divided into two parts. During the first month, the general bearing was assumed to be approximately 60°, followed by 2 months travelling due north. At a constant migration speed, this geometry means that the latitude increments were twice as large during the latter part of the trajectory in comparison to the early part.

### Influence of the lunar cycle

**Figure 4:**
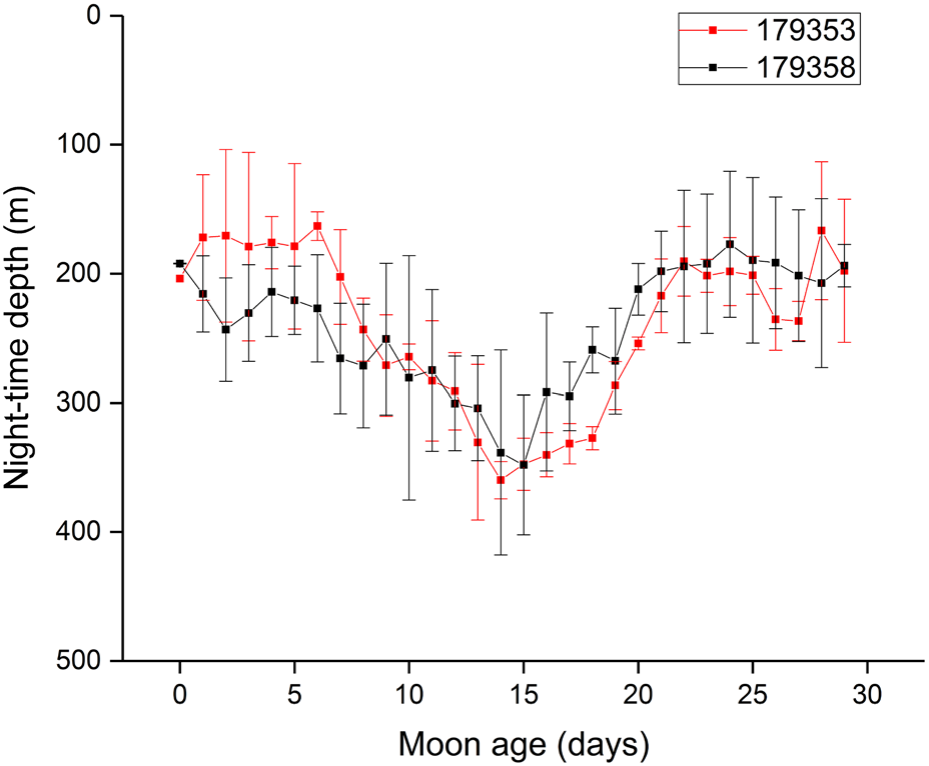
Night-time depth of the two longest tracks as a function of the age of the moon (new moon is at moon age 0, full moon is at age 14–15 days). The depth is measured over the period 12:00–15:00 UTC for a given day of the moon cycle and is averaged over 2–4 cycles for each eel.

The mean night-time swimming depth of all eels showing DVM varied in time with the phase of the moon, with the mean depth increasing with increasing moon irradiation. This is illustrated for the two eels with the longest tracks. The maximum depth was recorded 14 and 15 days after the new moon, which is the time of full moon (Fig. 4).

### Predation of eels

**Figure 5:**
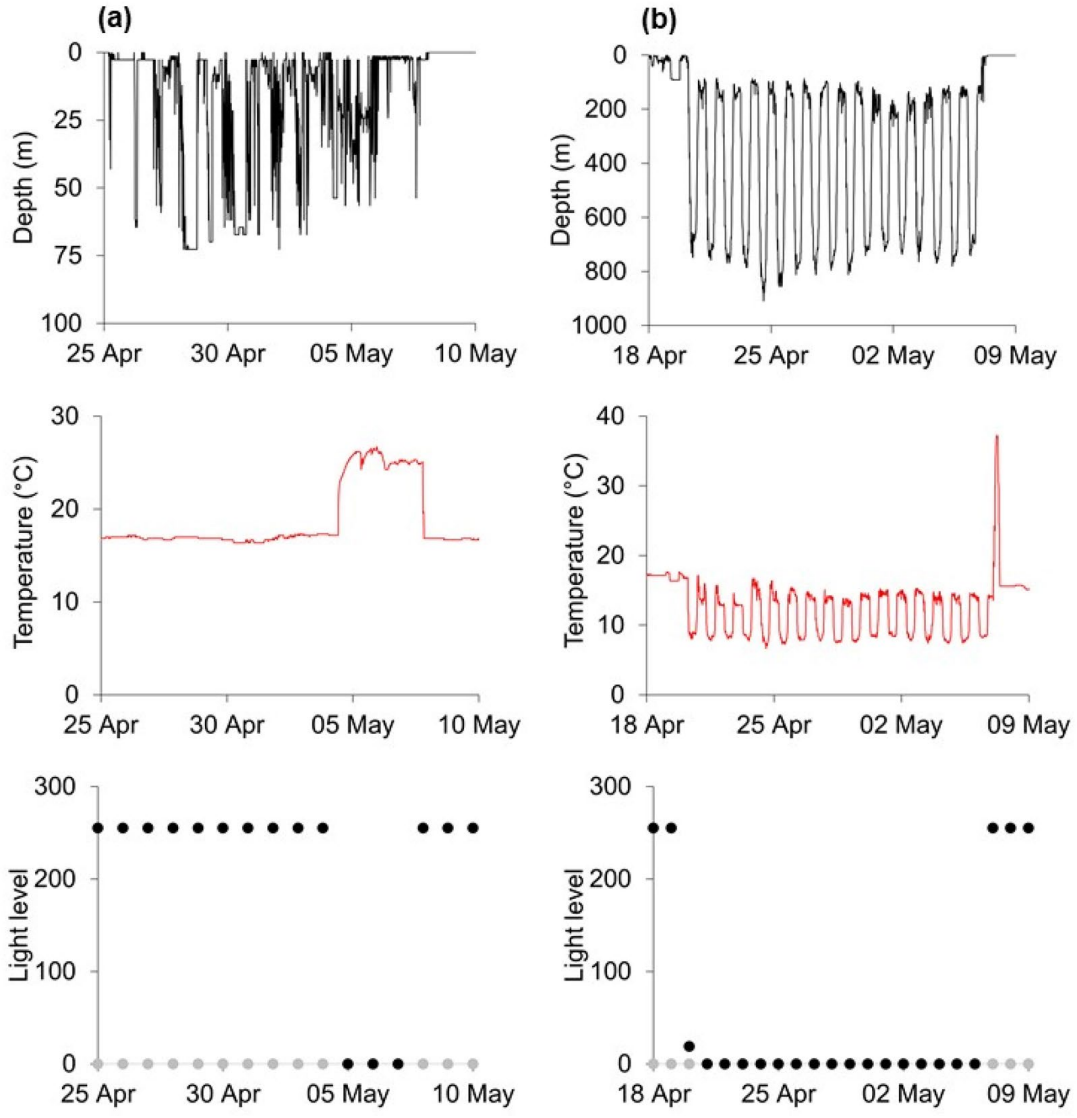
Examples of predation events for (**a**) eel 179386 in Bass Strait, showing a sudden temperature increase from ambient temperature to ~ 26 °C, indicative of a lamnid shark or a tuna, and (**b**) eel 179359 south of Tasmania, showing cessation of DVM behaviour during the day on 6 May 2019, at 150 m depth, and sudden temperature increase to approximately 37 °C, indicative of a marine mammal. Circles denote minimum (grey circle) and maximum (black circle) light level recorded.

Five tags (179353, 179357, 179359, 179360, 179386) were very likely ingested by predators (Fig. 1, Table 2), as demonstrated by a sudden temperature increase and lack of light recorded when the tag was close to the surface during day-time (i.e., the tag was inside the predator). Three PSATs remained within a predator for 4–8 days, where temperatures fluctuated between ~ 20 and 25 °C, indicative of lamnid sharks or tuna (Fig. 5). These three events occurred during the day, some 7–10 days following release, while the eels were negotiating the relatively shallow water of the Australian continental shelf. The other two eels appear to have been taken near the surface (150–170 m) at night by an animal with a stomach temperature of approximately 37 °C, indicating a marine mammal (e.g., whale) (Fig. 5).

**Figure 6:**
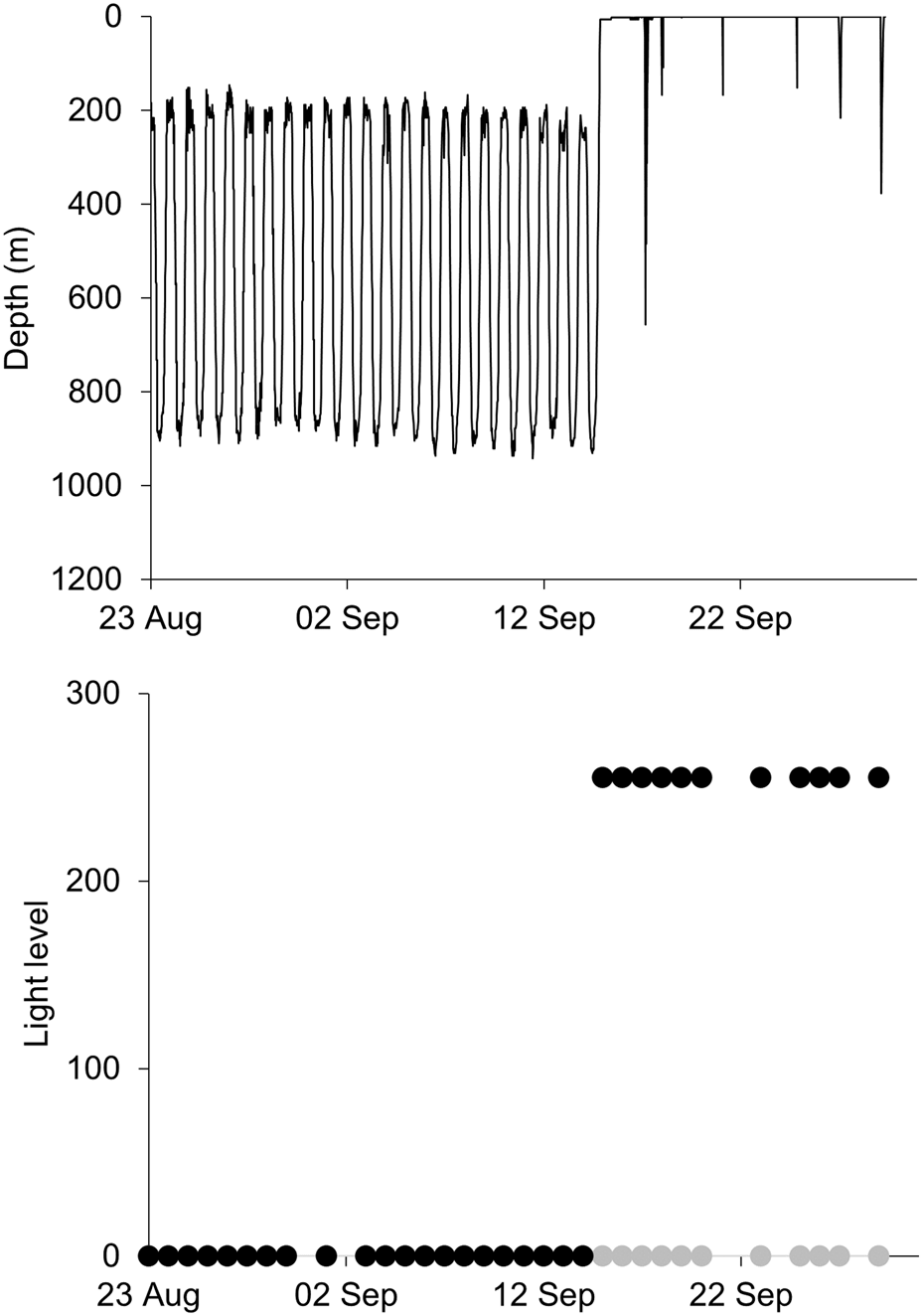
Example of a short-finned eel (179358) displaying regular DVM behaviour, followed by a rapid surface ascent and irregular dives from the surface. Circles denote minimum (grey circle) and maximum (black circle) light level recorded.

**Figure 7:**
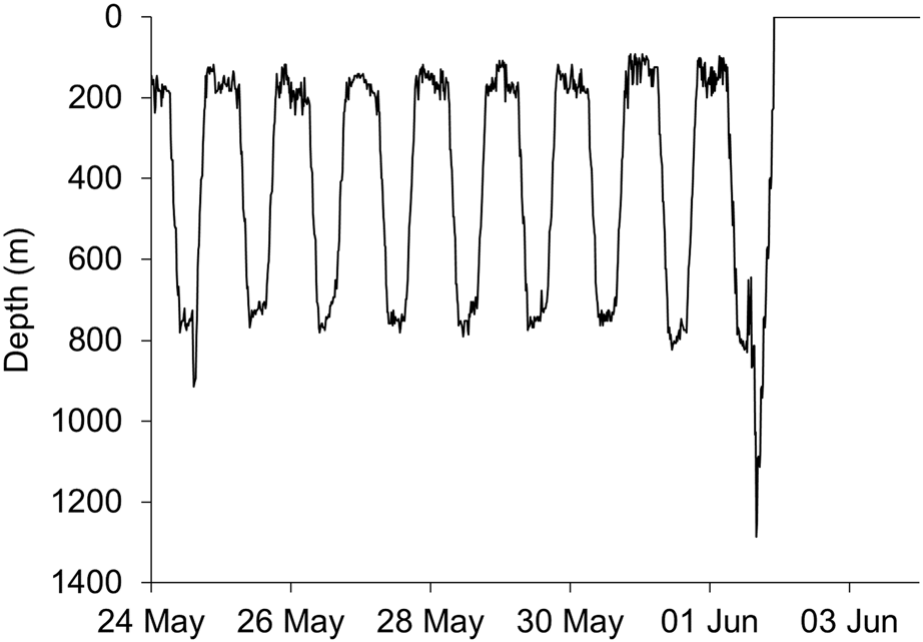
Example of a potential predation event of a short-finned eel (179385), showing a rapid dive from a normal 800-m depth DVM cycle to 1280 m, where the release mechanism was activated.

All tags released prematurely, and we cannot exclude the possibility that the remaining seven eels could also have been predated. Three tags (179352, 179358, 179361) changed from DVM to irregular dives from the surface, but the light sensor indicated that the tag was not ingested (Fig. 6). The cause of these irregular dives is unknown, and it is possible tags had detached from the eels at this stage, being pulled down by an unknown agent. Four tags suddenly rose to the surface, three (179354, 179363, 179364) during the DVM cycle in deep water. One of these (179385) was a rapid dive event to 1280 m, where the release mechanism was activated, and it may have been associated with a deep-ocean predation event (Fig. 7).

## Discussion

This study provides the first direct observations of the oceanic spawning migrations of Australasian short-finned eels. Tagged eels were tracked from the south-eastern coast of Australia for up to around 5 months and travelled up to ~ 2620 km from release and as far north as the tropical Coral Sea off the north-eastern coast of Australia. Two short-finned eels migrated to an area near New Caledonia, providing some support for the hypothesis of Schmidt^19^ that short-finned eels from Australia spawn on the western side of the New Caledonian submarine ridge. The timing of arrival at this region (July and September) also corresponds with estimated spawning times (July–October), based on back-calculated hatching dates of short-finned eel leptocephali from otolith analysis^27^.

### Migration routes

Due to limitations imposed by the data we received from the tags (absence of light or sea-surface temperature data due to the deep diving behaviour of the eels), we used a simple longitude-based method to reconstruct migration paths. While these reconstructions are somewhat uncertain, the reconstructed migration paths for the oceanic migration phase suggest trajectories to the east of the main core of the East Australian Current, which carries warm tropical waters from the Coral Sea southwards^32,33^. Swimming at great depth during the day means that the eels are moving in slower moving strata. The tagged short-finned eels also displayed individual variation in oceanic migration routes, and this was especially evident in the initial stages of migration. Four eels accessed deep water off the Australian continental shelf by swimming directly east through Bass Strait, while an equal number accessed deep water by swimming south-east and circumnavigating Tasmania. The mechanisms underlying this variation in oceanic route selection are unclear; however, different marine migration routes may reflect alternate recruitment routes of glass eels in the ocean, the imprinting of which might be used during the return journey to spawning areas^34^. An alternative hypothesis, as proposed for salmonids^35^, is that the adult eels follow the olfactory trail of juveniles. Migrating animals may sometimes travel off-course en route to their destination, indicating that they have only a crude map sense in the open ocean^36^. More detailed tracking of eels may reveal whether they similarly correct their course in the latter stages of migration.

### Predation of eels

Our study also provides important insights into interactions of short-finned eels with other predatory marine animals in near-shore and deep-sea marine environments. Predation appears to have ended many (at least ~ 30%) of the adult short-finned eel tracks in this study, corroborating previous studies reporting high (e.g., 25–85%) marine loss rates for anguillids^9,37,38^. Most of these tags were ingested and reported elevated temperatures relative to the ambient water temperature and up to 37 °C. These observations were consistent with the internal temperatures of endothermic predators such as lamnid sharks, tuna, or marine mammals. While PSATs conceivably increase predation susceptibility, a study in the Saint Lawrence River system, in Canada, also found low escapement rates of American eels internally tagged with small acoustic transmitters, linked to a high mortality rate possibly due to predation^39^. Thus, long-distance marine migrations are likely a risky component of eel life-history, as they are in the marine phase of other migratory fishes such as Atlantic salmon (*Salmo salar*)^40^. Increasingly, satellite tags are being used to infer mortality rates of fish and other taxa, including birds and turtles^41^, and they might similarly be used in eels to estimate mortality rate.

### Migration speed

The estimated speeds of migration for tagged short-finned eels (range 10–50 km/day) resembled estimates for other species (e.g. *A. anguilla*: 2–51 km/day^8,9,42^, *A. dieffenbachii*: 15–31 km/day^12^, *A. marmorata*: 9–23 km/day^30,43^, *A. megastoma*: 10–23 km/day^30,44^, and *A. rostrata*: 35–54 km/day^45^. However, as noted by Béguer-Pon et al.^45^, few studies have considered ocean currents, and this hampers comparisons of migration speed. Nevertheless, these results demonstrate that estimated migration speeds vary among individuals, suggesting that mature individuals likely arrive at the spawning areas over a broad time period. Indeed, for the two short-finned eels that reached the tropical Coral Sea, their timing of arrival (July and September) differed by several months, despite being released in the same location at the same time.

### Diel vertical migration

Short-finned eels exhibited regular DVM once in deep water. All anguillid species that have been studied using telemetry have shown the same large diurnal swimming depth cycle—shallow during the night and deeper during the day. Wu et al.^46^ compiled data from 11 studies on six species and reported the average night-time depth was 213 ± 88 m and the average day-time depth was 602 ± 150 m. The temperature range varied widely, depending on the conditions in the geographical area. For instance, in the Mediterranean the temperature during day and night was essentially the same, approximately 18 °C, and in the North Atlantic the minimum temperature during the day was close to 0 °C^9^. The data for short-finned eels falls within the range of the other species: moving from cooler (6–8 °C), deeper (700–900 m) environments during the day to warmer (15–20 °C), shallower (100–300 m) environments at night. It is well established that anguillid eels do not feed during their spawning migration^47^, so it has been suggested that the function of the vertical migrations relates to predator avoidance, swimming efficiency, thermal regulation, and control of maturation^9,45,48,49^. That the change in daylight intensity triggers the onset of ascent and descent seems to hold for all species, however.

### Influence of the moon

Short-finned eels occupied deeper water during nights with a full moon than when the moon phase was less than full, with a near linear relationship between moon age and night-time depth. This behaviour is consistent with other Pacific *Anguilla* species, such as *A. marmorata*^43^, *A. japonica*^10^, and *A. dieffenbachii*^13^, which tend to swim nearer the surface at night during the new moon than during full moon. However, this behaviour is not ubiquitous among anguillids, as no relationship between moon phase and swimming depth at night was observed for either *A. anguilla* or *A. rostrata*^45^. Lunar phases are a strong determinant of movement in many other animals, particularly through their influence on predator–prey interactions^50,51^. Our results suggest that short-finned eels are vulnerable to visual predators, and it seems likely therefore that they would be at a greater risk during fuller moon phases if they did not adjust their nocturnal swimming depth to reduce this risk. Outmigration of short-finned eels from the estuary into the sea is also strongly related to moon phase^52^, suggesting that moon phase is an important determinant of migration behaviour in this species.

### PSAT performance

In the present study, 11 of 16 (69%) tags transmitted their data to the Argos system. This proportion is broadly similar to the 79% reported in a meta-analysis of PSAT performance from the published literature^53^. The factors influencing PSAT transmission are not well known, although lower data capture rates are common for species that display deep-water (~ 1000 m) vertical migrations^53^. Numerous authors have suggested that reporting rates may be compromised by rapid changes in pressure or temperature associated with changes in depth^53,54^. We also found that damage to PSAT antennae following predation could preclude data transmission. Some of these issues reflect the intrinsically hostile environment where PSATs are required to function, while others may be resolved with further technology developments.

### Conservation management

Numerous anguillid species are under threat globally, and a better understanding of their life history, including their marine migrations, is needed. In relation to the conservation management of Australasian short-finned eels, our results show that eels from the species’ southern continental range undertake long-distance migrations to the tropical Coral Sea, although there is individual variation in oceanic migration routes to access deep water off the Australian coast. Such information on migratory routes could provide an important basis for informing future efforts to assess, prioritize, and mitigate potential interactions between eels and human activities in both the freshwater and marine environments. Anthropogenic factors in the marine environment, such as deep-sea mining, and the construction and operation of energy developments, for instance, may interact with migrating *Anguilla* species^55^.

Our results also indicate that oceanic predation is likely to be a strong regulator of the number of adult eels escaping the continent and reaching their spawning grounds. Such information on eel mortality has the potential, for example, to better inform stock assessments used to determine fishery quotas^56,57^, since stock assessments and models are based on the number of eels that leave the river rather than the number of eels that reach their spawning grounds^58,59^. This has important consequences for the accuracy of assessments and predictive models. Over coming years, developing a strong predictive modeling capacity for managing short-finned eel populations in freshwater, estuarine, and marine environments is especially important against the stark backdrop of global population declines.

## Supplementary Information

Supplementary Information.

## Data Availability

The datasets generated during the current study are available from the corresponding author on reasonable request.
