# Supplementary material for: First tracking of the oceanic spawning migrations of Australasian short-finned eels (Anguilla australis)
Source: Sci Rep. 2021 Nov 26;11:22976. doi: 10.1038/s41598-021-02325-9 (PMC8626517; doi:10.1038/s41598-021-02325-9)
Supplement: Supplementary file 1 — Supplementary Information. [file 41598_2021_2325_MOESM1_ESM.docx]

**Supplementary Information**

First tracking of the oceanic spawning migrations of Australasian short-finned eels (*Anguilla australis*)

Wayne M. Koster^1*^, Kim Aarestrup^2^, Kim Birnie-Gauvin^2^, Ben Church^3^, David Dawson^1^, Jarod Lyon^1^, Justin O’Connor^1^, David Righton^4,5^, Denis Rose^3^, Håkan Westerberg^6^ & Ivor Stuart^1^

Estimates of the daily longitude along the trajectory of migrating anguillid eels are based on the assumption that the diel vertical migration mirrors the daylight cycle[1]. This principle has been demonstrated for Atlantic eels (e.g. Righton et al.[2] on *A. anguilla*; and Béguer-Pon et al.[3] on *A. rostrata*). In both cases, the times when the tagged eel crossed a reference depth were used to approximate the times of dawn and dusk, and hence to calculate the time of local noon. This approach relies on two properties of the depth time series; first, that the series of observed values are recorded with sufficient frequency to obtain accurate estimates of the time of dawn and dusk and, second, that the rate of vertical movement is not significantly faster than the logging capacity of the archival tag. In the case of *A. australis*, the time series values received by satellite transmission were sufficiently frequent but, critically, the rate of vertical movement (being approximately twice that observed for Atlantic eels) means that the X-tag data compression technology replaces the true values of depth during the steep dawn and dusk periods with estimated values, a process known as delta-limitation. Delta-limitation is a property of the data compression method used for the X-tag, in which true depth differences between measurements are replaced by a value limited by the digitalization of the actual measurement values. A detailed explanation of the process of data compression and delta-limitation can be found at https://www.microwavetelemetry.com/compression_techniques_used_in_standard_rate_tags?s=delta%20limit. For this reason, using a reference depth method of estimation for local noon proved to give very poor results for the short-finned eel data (Figure S1), because the differences between delta-limited, successive depth values will be smaller than the true values, and the estimate of the midpoint of the DVM cycle will be delayed in time at both dawn and dusk; the result is a longitude estimate with an error toward the east.

**Figure S1.** Last day before pop-up of tag 179385. Red dots represent actual values; blue triangles represent delta-limited values. The actual and estimated times of sunrise and sunset are indicated using a fixed reference level (average daily swimming depth) and the empirical approach following Chang et al.[4].

An alternative to a fixed reference depth for estimating sunrise and sunset times is the approach used by Chang et al.[4] for reconstructing the tracks of *Anguilla marmorata*. They used an empirical approach in which the observed DVM cycle close to the pop-up position was compared with the time of sunrise and sunset at this position and date. They determined the time of sunset as the beginning of the steep positive depth gradient, and the time of sunrise as the time of the median of the steep negative gradient. A similar analysis of the *A. australis* data also shows that sunset in many cases coincides with the start of the steep rise of the eel and that the sunrise occurs approximately when the delta-limited depth values cross the daily mean swimming depth (Figure S1).

The explanation of the asymmetry is that the trigger for ascent and descent takes place at a constant daylight irradiance. At sunset, the eel is deep in the water column (where the light extinction is greater), and therefore the critical light level will be reached earlier than at the reference depth, before the sun disappears below the horizon. In contrast, at sunrise the eel is relatively shallow and the same critical light level is reached earlier than at the reference depth, when the sun is below the horizon but the twilight is increasing. Hence the estimated timings of dusk and dawn need to be taken from different phases of the large vertical ascents to offset the effects of light extinction.

Since it can be difficult to ascertain with certainty when the start of the large ascent occurs, we used a simplified version of the Chang et al. method[4], in which the time of the last actual depth value (i.e., not delta) before the delta-limited steep ascent was taken as the time of sunset, and the time of the delta-limited depth value close to the mean daily swimming depth during the steep descent was taken as a proxy for the time of sunrise. Fig S2 compares longitude estimates of the trajectory of eel 179358 using the fixed reference level with this empirical method.

**Figure S2.** Comparison of daily longitude estimates of eel 179358, using the empirical approach following Chang et al.[4] (red dots) and estimates based on crossing of a fixed reference level (blue dots). Red and blue lines are daily data smoothed with a locally weighted scatterplot smoothing (LOWESS) over a 13-points span.

**References**

1. Westerberg, H. Marine migratory behavior of the European silver eel. In *Physiology and Ecology of Fish Migration* (eds H. Ueda, H. & Tsukamoto, K.) 80–103 (CRC Press, 2013).

2. Righton, D. *et al*. Empirical observations of the spawning migration of European eels: the long and dangerous road to the Sargasso Sea. *Sci. Adv.* **2,** e1501694 (2016).

3. Béguer-Pon, M., Shan, S., Castonguay, M. & Dodson, J. J. Behavioural variability in the vertical and horizontal oceanic migrations of silver American eels. *Mar. Ecol. Prog. Ser.* **585,** 123–142 (2017).

4. Chang, Y.-L. K., Olmo, G. D. & Schabetsberger, R. Tracking the marine migration routes of South Pacific silver eels. *Mar. Ecol. Prog. Ser.* **646,** 1–12 (2020).
